# The Relationship of Oral Health between Psychological States in Adults of South Korea

**Published:** 2019-02

**Authors:** Yong-Keum CHOI, Yoonhee KIM, Jung-Hui SON, Jin-Sun CHOI

**Affiliations:** Department of Preventive and Public Health Dentistry, Gangneung-Wonju National University, 7 Jukheongil, Gangneung, Ganwondo 25457, Republic of Korea

## Dear Editor-in-Chief

Oral health plays an important role in maintaining the quality of life of the individual’s psychological health (1). To confirm the relationship oral health between psychological states, the difference of satisfaction according to the degree of depression was investigated, and the subjects with severe depression were more dissatisfied with the full denture (2). There is also a cross-sectional study of the relationship between anxiety, depression and oral health. In this study, the higher the frequency of anxiety and depression gum bleeding, and toothache were 2.9 times higher than normal subjects (3).

Psychological states are emerging as one of the important health problems of modern people as societies change and become increasingly complex, and these psychological states are closely related to oral health (4, 5).

However, there is a lack of research on the relationship between the psychological states and disability of mastication and speaking functional cognition of subjects who are subjective indicators of oral health. This study aimed to determine the oral masticatory function among mental health in over 20-yr-old South Korean.

The 2007 Korea National Health and Nutrition Survey (KNHANES) had the complex survey design which considers non-response rates and post-stratification adjustment to match Korean population by gender and age structures (Table 1).

**Table 1:** Distribution of demographic characteristics, oral health and mental health status

***Characteristics***	***No. of Subjects (n=3335)***	***Weighted percent (SE)***
Sex		
Male	1,242	49.3 (0.8)
Female	1,741	50.7 (0.8)
Age group		
20–40	974	43.6 (1.4)
40–64	1,287	43.4 (1.4)
65≤	722	13.0 (0.8)
TMD (Temporomandibular Disorder)		
Yes	82	3.2 (0.4)
No	2,901	96.8 (0.4)
Income (household)		
1^st^ quantile	631	16.1 (1.5)
2^nd^ quantile	742	26.1 (1.7)
3^rd^ quantile	726	28.6 (1.4)
4^th^ quantile	745	29.2 (2.2)
Disability of Mastication		
Yes	1,036	29.2 (1)
No	1,942	70.8 (1)
Disability of Speaking		
Yes	375	9.9 (0.8)
No	2,601	90.1 (0.8)
The amount of stress		
High	780	27.1 (0.9)
Low	2,198	72.9 (0.9)
Depression during recent 2wk		
Yes	436	12.7 (0.6)
No	2,544	87.3 (0.6)
Impulse of suicide during recent a year		
Yes	521	15.0 (0.9)
No	2,452	85.0 (0.9)
Prosthodontic status (maxillar)		
Sound teeth or filling or single crown	1,841	70.8 (0.9)
One bridge	481	15.5 (0.7)
Over two bridges	215	5.5 (0.4)
Only partial denture	91	1.8 (0.2)
Bridges and partial denture	109	2.5 (0.3)
Full denture	188	3.8 (0.4)
Prosthodontic status (mandibular)		
Sound teeth or filling or single crown	1,898	73.4 (0.9)
One bridge	394	11.6 (0.7)
Over two bridges	252	6.7 (0.5)
Only partial denture	112	2.2 (0.3)
Bridges and partial denture	151	3.8 (0.4)
Full denture	118	2.3 (0.4)

The logistic regression model was used to estimate the association between psychological problems and oral discomforts. In particular, we used PROC SURVEYLOGISTIC procedure of SAS version 9.1 due to the reason why the KNHANES data were collected from National Survey program designed as complex sampling. For statistical inferences, PROC SURVEYLOGISTIC incorporates complex survey sample designs, including designs with stratification, clustering, and unequal weighting.

Table 2 shows the relationships between the psychological status and disabilities of speaking and mastication, respectively.

**Table 2:** Relationship between the phycological states and disability of mastication and speaking OR (95% CI)

***Predictors***	***Disability of mastication***			***Disability of speaking***		
	***Amount of stress***	***Depression during Recent 2 wk***	***Impulse of suicide during Recent a year***	***Amount of stress***	***Depression during Recent 2 wk***	***Impulse of Suicide during Recent a year***
Sex						
Male	1.00	1.00	1.00	1.00	1.00	1.00
Female	1.4(1.14–1.73)	2.41 (1.83–3.16)	2.53 (1.93–3.33)	1.4 (1.13–1.73)	2.39 (1.83–3.13)	2.5 (1.9–3.27)
Age group						
20–40	1.00	1.00	1.00	1.00	1.00	1.00
40–64	0.9(0.69–1.18)	1.32 (0.94–1.87)	1.2 (0.87–1.64)	0.92 (0.7–1.2)	1.36 (0.97–1.9)	1.26 (0.92–1.74)
65≤	0.8(0.56–1.14)	1.72 (1.16–2.56)	2.21 (1.53–3.19)	0.8 (0.56–1.15)	1.69 (1.16–2.46)	2.34 (1.61–3.4)
TMD^*^						
Yes	1.89(1.15–3.11)	1.34 (0.66–2.72)	2.1 (1.04–4.22)	1.93 (1.16–3.21)	1.36 (0.66–2.79)	2.13 (1.03–4.38)
No	1.00	1.00	1.00	1.00	1.00	1.00
Income (household)						
1^st^quantile	1.17(0.78–1.76)	2 (1.41–2.83)	2.21 (1.41–3.44)	1.19 (0.79–1.8)	2.05 (1.44–2.94)	2.36 (1.51–3.69)
2^nd^quantile	1.21(0.91–1.61)	1.52 (1.08–2.14)	2.29 (1.55–3.38)	1.24 (0.93–1.65)	1.58 (1.11–2.26)	2.42 (1.64–3.59)
3^rd^quantile	1.14(0.88–1.49)	1.36 (0.94–1.96)	1.01 (0.67–1.51)	1.16 (0.89–1.5)	1.39 (0.97–2)	1.04 (0.7–1.56)
4^th^quantile	1.00	1.00	1.00	1.00	1.00	1.00
Disability of Speaking						
Yes	1.33(1.09–1.62)	1.64 (1.21–2.24)	1.82 (1.37–2.42)	1.54 (1.19–1.98)	2.17 (1.65–2.86)	1.87 (1.38–2.55)
No	1.00	1.00	1.00	1.00	1.00	1.00

* TMD (Temporomandibular Disorder)

The people with disability of speaking had a 1.33 times higher likelihood of perceived high amount of stress, 1.64 higher of the depression during recent 2 wk, and a 1.82 higher of the impulse of suicide during recent a year than normal subjects. Moreover, the people with disability of mastication had a 1.54 times higher likelihood of perceived high amount of stress, a 2.17 higher of the depression during recent 2 wk, and a 1.87 higher of the impulse of suicide during recent a year than normal subjects.

There was a disability of mastication and speaking affect depression. Maintaining oral health will help you improvement psychological states. In other words, keeping oral health, trying to avoid mastication and speaking problems will help improve mental health. Conversely, when consulting for mental problems or developing a program to improve mental health, it may be helpful to verify that there is no oral health or mastication and speaking problems.

## References

[1] McGrath C Bedi R (2001). An evaluation of a new measure of oral health-related quality of life OHQol-UK(W). Community Dent Health, 18(3):138–143. 11580088

[2] Elter JR White BA Gaynes BN Bader JD (2002). Relationship of clinical depression to periodontal treatment outcome. J Periodontol, 73(4):441–9. 11990446 10.1902/jop.2002.73.4.441

[3] Marques Vidal P Milagre V (2006). Are oral health status and care associated with anxiety and depression? A study of Portuguese Health science students. J Public Health Dent, 66(1):64–6. 16570753 10.1111/j.1752-7325.2006.tb02553.x

[4] John MT Micheelis W Steele JG (2007). Depression as a risk factor for denture dissatisfaction. J Dent Res, 86(9):852–6. 17720854 10.1177/154405910708600909

[5] Gatchel RJ Stowell AW Buschang P (2006). The relationships among depression, pain, and masticatory functioning in temporomandibular disorder patients. J Orofac Pain, 20(4):288–96. 17190027

